# The Impact of the Early COVID-19 Pandemic on ST-Segment Elevation Myocardial Infarction Presentation and Outcomes—A Systematic Review and Meta-Analysis

**DOI:** 10.3390/diagnostics12030588

**Published:** 2022-02-25

**Authors:** Cristina Furnica, Raluca Ozana Chistol, Dragos Andrei Chiran, Cristinel Ionel Stan, Gabriela Dumachita Sargu, Nona Girlescu, Grigore Tinica

**Affiliations:** 1 Faculty of Medicine, “Grigore T. Popa” University of Medicine and Pharmacy, 700115 Iasi, Romania; cristina.furnica@umfiasi.ro (C.F.); cristinel.stan@umfiasi.ro (C.I.S.); sargu_gabriela@yahoo.com (G.D.S.); nona-girlescu@umfiasi.ro (N.G.); grigore.tinica@umfiasi.ro (G.T.); 2 Institute of Forensic Medicine, 700455 Iasi, Romania; 3 “Prof. Dr. George I.M. Georgescu” Cardiovascular Diseases Institute, 700503 Iasi, Romania; 4 “Elena Doamna” Obstetrics and Gynecology Hospital, 700398 Iasi, Romania

**Keywords:** symptoms-to-first-medical-contact time, door-to-balloon time, total ischemic time, left ventricular ejection fraction, troponin I, mortality

## Abstract

Background: The influence of the early COVID-19 pandemic on non-COVID-19 emergencies is uncertain. We conducted a systematic review and a meta-analysis to evaluate the impact of the first months of the COVID-19 pandemic on the presentation, management, and prognosis of patients presenting with ST-segment elevation myocardial infarction (STEMI). Methods: We searched the PubMed, Scopus, and Embase databases from January to August 2020. A meta-analysis of studies comparing the profile, STEMI severity at presentation, reperfusion delay, and in-hospital mortality for patients presenting before and during the early COVID-19 pandemic was conducted. Fifteen cross-sectional observational studies including 20,528 STEMI patients from the pre-COVID period and 2190 patients diagnosed and treated during the first months of the COVID-19 pandemic met the inclusion criteria. Results: Patients presenting with STEMI during the pandemic were younger and had a higher comorbidity burden. The time interval between symptoms and first medical contact increased from 93.22 ± 137.37 min to 142 ± 281.60 min (*p* < 0.001). Door-to-balloon time did not differ significantly between the two periods (*p* = 0.293). The pooled odds ratio (OR) for low left ventricular ejection fraction at presentation during the pandemic was 2.24 (95% confidence interval (CI) 1.54–3.26) and for a presentation delay >24 h was 2.9 (95% CI 1.54–5.45) relative to before the pandemic. In-hospital mortality did not increase significantly during the outbreak (*p* = 0.97). Conclusion: During the first months of the COVID-19 pandemic, patients presenting with STEMI were addressed later in the course of the disease with more severe left ventricular impairment. In-hospital emergency circuits and care functioned properly with no increase in door-to-balloon time and early mortality.

## 1. Introduction

The new severe acute respiratory syndrome coronavirus-2 (SARS-CoV-2) that was isolated in the Chinese city of Wuhan in December 2019 caught the entire world by surprise and rapidly evolved to a pandemic. Medical systems worldwide were forced to adopt measures to prevent the spread of the coronavirus disease (COVID-19). The penetrance of the virus varied among different countries around the world, with China and Italy being the most affected in the initial phase of the pandemic [1]. Authorities all around the world issued new laws and recommendations restricting certain rights and freedoms (movement, travel), limiting social interaction, and imposing self-confinement. Therefore, medical systems were also redesigned, with certain facilities being transformed into COVID-19 hospitals, new triage systems, patient separation, limitation of presentations in an outpatient setting, and novel management of emergencies. Patients became more reluctant to seek medical help because of fear of viral contamination, confusion with COVID-19 symptoms [2], lack of correct information concerning the state of medical services, and lockdown measures.

Non-COVID-19 emergencies were also affected by the pandemic. Reports as well as unicentric and multicentric studies were published in a short period by teams from various countries to describe the experience with acute coronary syndromes presentation, management, and prognosis during periods of restrictive social and medical measures. Because of the absence of a clear image of the influence of the first months of the COVID-19 pandemic on patients with ST-segment elevation myocardial infarction (STEMI), we conducted a systematic review of the literature and a meta-analysis of selected studies to evaluate the impact of the early COVID-19 pandemic on the presentation, management, and prognosis of STEMI patients.

## 2. Materials and Methods

The international prospective register of systematic reviews (PROSPERO) registration number for this study is: CRD42020202468.

### 2.1. Search Strategy

**Figure 1:**
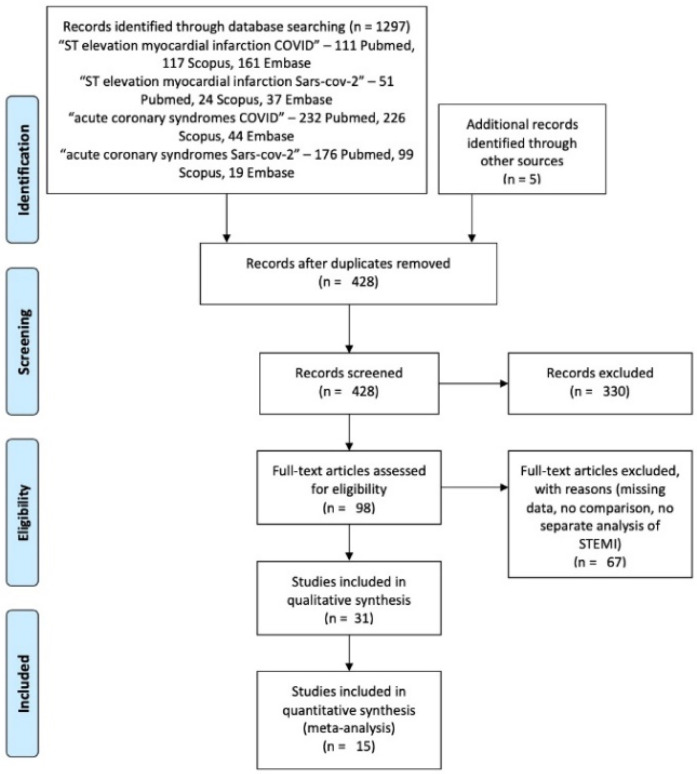
PRISMA flow chart of the selection process.

PubMed, Scopus, and Embase databases were searched using the following queries: ST elevation myocardial infarction COVID, ST elevation myocardial infarction SARS-CoV-2, acute coronary syndromes COVID, and acute coronary syndromes SARS-CoV-2—returning the results displayed in Figure 1. No language limitation was applied. Identified references were checked for duplicates and a total of 428 records resulted. All abstracts were screened manually for additional removal of 330 reviews, letters to the editor, editorials or short communications containing no actual data. A total of 98 abstracts qualified for further evaluation of the full-text article to decide whether they met the inclusion and exclusion criteria. Another 67 articles were excluded due to missing data, absence of a control group, or no separate analysis of STEMI. Finally, 15 studies were included in the quantitative synthesis (Table 1).

### 2.2. Inclusion and Exclusion Criteria

Included studies met the following criteria: (1) the study focused on the impact of the first months of the COVID-19 pandemic on patients presenting with STEMI regarding reperfusion delay (symptom-to-first-medical-contact (FMC) time, door-to-balloon time, total ischemic time, presentation > 24 h), STEMI severity at presentation (LVEF, cTn-I), and in-hospital mortality; (2) the study was a cross-sectional observational study (prospective or retrospective) reporting data from a lockdown period or a period of maximal social/health care measures in the respective country (e.g., stop of elective procedures); (3) the study provided the number of cases, means, and standard deviations or sufficient information to calculate them.

A study was excluded from the meta-analysis if it (1) only provided information from the period associated to the early COVID-19 pandemic and no comparison with a pre-COVID-19 control group from the same institution(s); (2) presented only percentages, percentage differences, or mean values differences; (3) was a low-quality investigation.

### 2.3. Data Extraction

Three investigators (G.D.S., C.I.S., and N.G.) extracted the following data from each selected study: first author’s surname, number of centres that provided data for the study, the country the study was conducted in, sample size ((1) if the study analysed the whole spectrum of ACS data was extracted for STEMI only; (2) if the study also contained SARS-CoV-2 positive patients, mortality was assessed only for SARS-CoV-2 negative patients), baseline data, and outcome data. A 4th investigator, G.T., analysed the final data and referred to the original article in case of difference between extracted data. When incomplete data were provided, the corresponding author was contacted.

### 2.4. Quality Assessment

The Newcastle–Ottawa Scale (NOS) for assessing the quality of nonrandomised studies in meta-analyses was used to evaluate the quality of the studies included. All studies scored 6 or 7 stars (high-quality studies) (Table 2). Two investigators independently evaluated the selected articles (G.T. and R.O.C.).

### 2.5. Statistical Analysis

Studies included in the analysis were functionally identical (cross-sectional observational), with the effect size differing mainly because of sampling. We used the Review Manager (RevMan) 5.4 (The Cochrane Collaboration, 2020) software to compute the pooled effect size with mean difference and 95% confidence intervals (CI) by the inverse variance method for continuous variables, and with odds ratio (OR) and 95% CI by the Mantel–Haenszel method for dichotomous variables. The heterogeneity among studies was estimated by a chi-squared-based Q test and I2 statistics; a *p* value > 0.05 for the Q test and an I2 > 50% was considered a measure of important heterogeneity. When heterogeneity was found across the analysis, the random effect method was used for analysing the data. Online supplements were consulted, and the authors used them to complete the data when possible. The pooled sample mean and pooled standard deviation (SD) were calculated according to the recommendations of the Cochrane Handbook of Systematic Reviews [17]. When median and interquartile ranges were reported, mean and standard deviation were estimated using the methods described by Luo et al. [18] and Wan et al. [19], respectively. When mean was reported together with 95% CI, RevMan Calculator (Cochrane Training) was used to compute standard deviation. Mortality and STEMI severity evaluation (using left ventricular ejection fraction (LVEF) and troponin I (cTn-I) levels at presentation) were evaluated only for SARS-CoV-2-negative patients presenting during the COVID-19 pandemic. Three of the selected studies also included SARS-CoV-2-positive patients. For these studies, the mortality of SARS-CoV-2-negative patients was recalculated using available data. Publication bias was assessed using the funnel plot.

## 3. Results

The 15 selected studies included a total of 20,528 STEMI patients diagnosed and treated in the pre-COVID period and 2190 patients diagnosed and treated during a period of restrictions because of the COVID-19 pandemic.

### 3.1. Patients’ Profile

The baseline characteristics of patients from the groups studied are reported in Table 3.

Patients presenting with STEMI during the first months of the COVID-19 pandemic were younger and registered a higher incidence of CAD risk factors compared to patients presenting before the pandemic.

### 3.2. STEMI Presentation

**Figure 2:**
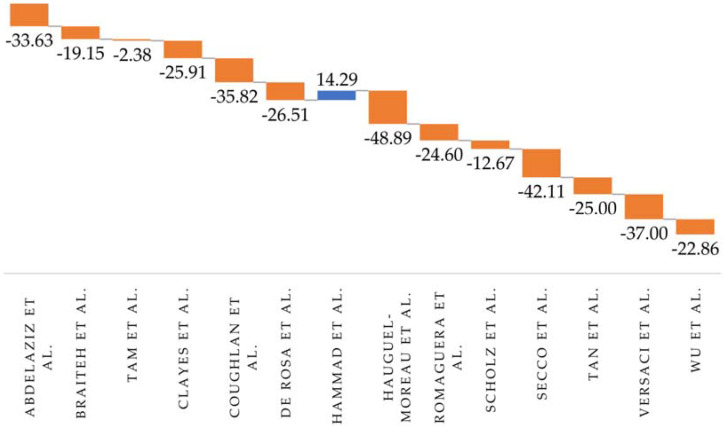
Percentage variation of the number of patients presenting daily during the pandemic compared to the period before.

A decrease in the number of STEMI patients presenting daily was identified and quantified using data from 13 studies, with decreases ranging from 2.38% to 48.89%. Hammad et al. [2] were the only ones to signal an 14.29% increase in presentations (35 patients over a 23-day period during the COVID-19 pandemic compared to 108 patients over an 81-day period before). In the case of Popovic et al. [10], the number of cases/day could not be estimated given the imprecise “before” interval (cohort of STEMI patients treated during 2008–2017) (Figure 2).

**Figure 3:**
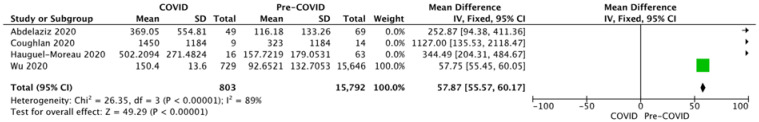
Forest plots for symptoms-to-FMC time.

The time interval between symptoms and the first medical contact (FMC) was reported by five studies but in the pooled analysis only four were included. The research of Scholz et al. [12] was eliminated from the overall estimate as they analysed patients from the FITT-STEMI study which includes only cases presenting within 24 h from symptom onset. Pooled results of the four remaining studies revealed that in the pre-COVID-19 period, patients were addressed at 93.22 ± 137.37 min after the onset of symptoms compared to 142 ± 281.60 min during the first months of the pandemic (*p* < 0.001); according to the forest plot, however, there is great heterogeneity across the studies (I2 = 88%, *p* < 0.01) for reported data, and we could not calculate a summary effect (Figure 3).

**Figure 4:**
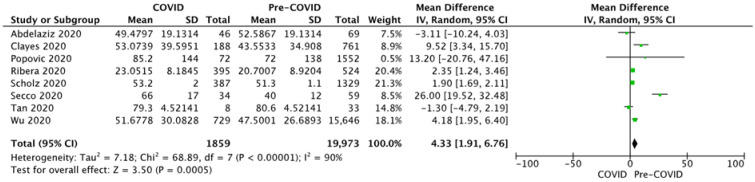
Forest plots for door-to-balloon time.

Door-to-balloon time was reported by eight studies and did not differ significantly between the two periods (48.85 ± 46.42 min before compared to 47.68 ± 39.29 min during the pandemic, *p* = 0.293). Similar to symptoms-to-FMC time, the forest plot indicated a significant heterogeneity across the studies (I2 = 90%, *p* < 0.001), but the pooled effect was balanced compared to the previous case (Figure 4).

**Figure 5:**
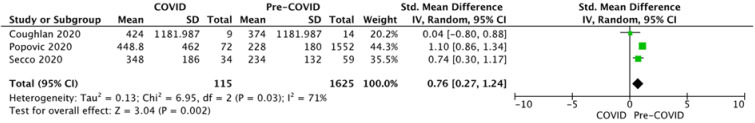
Forest plots for total ischemic time.

The total ischemic time was extracted from three studies, and standard mean difference was used to assess the pooled effect as for Coughlan et al. [7] the SD was computed from reported statistics (mean, 95% CI and *p* value). The mean total ischemia time increased from 229.48 ± 207.20 min to 417.06 ± 493.004 min during the pandemic (*p* < 0.001). A significant heterogeneity was found across the studies (I2 = 71%, *p* < 0.002), which again did not allow us to calculate a summary effect (Figure 5).

**Figure 6:**
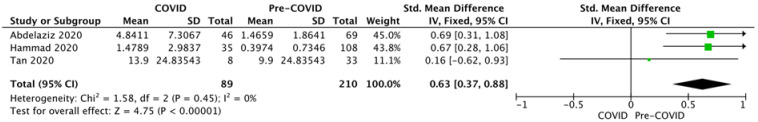
Forest plots for troponin-I level.

The troponin-I level (cTn-I) was reported by three studies, and standard mean difference was used to assess the pooled effect as studies used different measurement methods and laboratory reference values. Irrespective to the measurement method, a higher troponin level was registered at admission during the COVID-19 pandemic (Figure 6) with no heterogeneity across the studies (I2 = 0%, *p* = 0.45).

**Figure 7:**
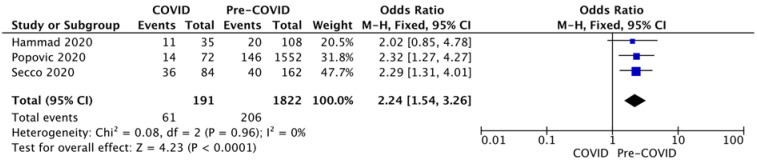
Forest plots for LVEF at presentation.

The percentage of patients with a low LVEF (<40%) at presentation was reported with no heterogeneity (I2 = 0%, *p* = 0.96) by three studies that were unanimous in finding an association between the presentation during the COVID-19 outbreak and the severity of the myocardial infarction. The pooled analysis of the three studies revealed a significant increase in severe left ventricular impairment risk by a factor of 2.24 in a fixed-effects model (Figure 7).

**Figure 8:**
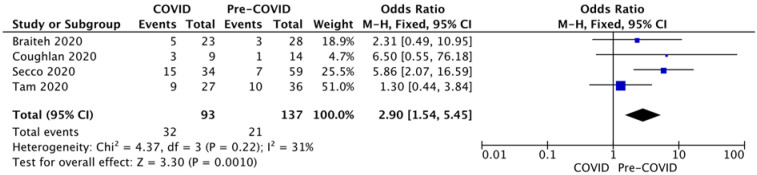
Forest plots for presentation delay >24 h.

A presentation delay beyond 24 h was reported by four studies with low heterogeneity (I2 = 31%, *p* = 0.22) with two studies only reporting the delay >12 h. Pooled analysis of the four studies identified a significant increase in late presentation risk during the outbreak by a factor of 2.9 (Figure 8).

### 3.3. STEMI Outcome

**Figure 9:**
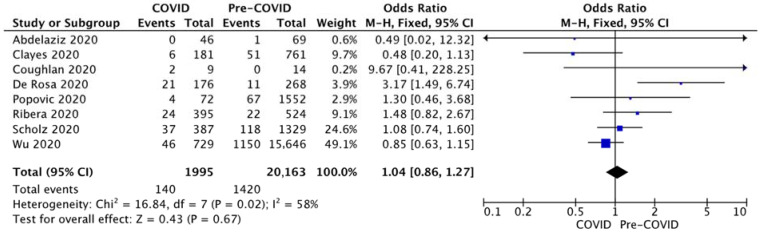
Forest plots for In-hospital mortality.

In-hospital mortality was reported by eight studies with increased heterogeneity (I2 = 58%, *p* = 0.02) and the pooled effect did not reveal a significant increase. Overall, in-hospital mortality was 7.04% for STEMI patients presenting before the pandemic compared to 7.02% during the first months of the pandemic (*p* = 0.97). The pooled effect is largely based on the mortality reported by three large multicentric studies [11,12,16] contributing to more than 80% of the total weight. The funnel plot did not reveal a significant bias (Figure 9).

## 4. Discussion

### 4.1. Patients’ Profile

This meta-analysis is the first to evaluate the impact of the first months of the COVID-19 pandemic on the presentation and outcome of STEMI patients. Our results suggest that patients presenting with STEMI during the pandemic are younger and register a higher comorbidity burden for acute coronary syndromes (arterial hypertension, diabetes mellitus, smoking, dyslipidaemia, and known coronary artery disease). From the 31 studies included in the qualitative synthesis, only 3 [2,14,20] reported that patients presenting during the COVID-19 period were older. Irrespective of age, the studies were unanimous in quantifying a higher prevalence of comorbid statuses in patients with STEMI during the pandemic. Interestingly, ACS and SARS-CoV-2 share the same risk profile: patients most likely to present with STEMI are those at a higher risk of complications in case of SARS-CoV-2 infection.

### 4.2. STEMI Presentation

A decline in the admission rate was quantified using data from 13 of the 15 studies, with percentages ranging from 2.38% in the study of Tam et al. [5] to a maximum of 65% in the research of Hauguel-Moreau et al. [9]. Hammad et al. [2] were the only ones to report a 14.29% increase in their multicentric study performed in the USA, and Rattka et al. [21] to report an unchanged addressability. The decreasing trend was registered irrespective of the penetrance of SARS-CoV-2 in the country in which the research was performed. This indicates that patients might have avoided contact with healthcare systems worldwide because of fear of infection, new emergency response measures, strict guidelines for self-isolation during lockdown periods, and social distancing. Governmental calls to avoid overloading healthcare facilities and to seek medical care only in cases of emergencies were suspected as a concurrent factor by Rattka et al. [21]. Moreover, primary care physicians restricted their activity during the COVID-19 outbreak in several countries, such as France [22]. Consequently, there have could been STEMI cases that never reached the hospital [8,10,16,23]. A true reduction in the incidence of ACS was indicated as a potential additional explanation by several studies [4,6,15,24] because of limited physical activity, work from home, lack of environmental triggers, less smoking, better medication adherence, and lower pollution levels. An interesting factor was signalled by Hauguel-Moreau et al. and Kessler et al.—avoidance of medical care by altruism [9,25].

An increase in symptoms-to-FMC time was identified in four studies [3,7,9,16] and a slight decrease in the study of Scholz et al. [12] as they only included patients presenting within 24 h from symptom onset. The studies were heterogenous in reporting the time intervals and we computed a mean 142 ± 281.60 min delay during the pandemic compared to 93.22 ± 137.37 min before. This aspect is extremely important, as Brodie et al. proved that patients with symptoms-to-FMC time <90 min experience a reduction in 1-year mortality. The highest increase in symptoms-to-FMC time was found in the study of Coughlan et al. [7], from 323 to 1450 min, followed by that of Hauguel-Moreau et al. [9], from 121 to 600 min (median values). In another study not included in our meta-analysis, Wilson et al. [26] indicate a three-time increase in symptoms-to-FMC time. The same fear of contamination and misinterpretation of symptoms like cough and shortness of breath (assimilated to SARS-CoV-2 infection) were considered trigger factors by Firouzi et al., Braiteh et al., and Rattka et al. [4,21,27].

The door-to-balloon time was reported by eight studies and did not register a significant change during the first months of the COVID-19 pandemic (to 47.68 ± 39.29 min vs. 48.85 ± 46.42 min before). The only study that reported a significant increase from 39 to 45 min was the one performed by Clayes et al. [6]. Compared to symptoms-to-FMC time, door-to-balloon time is under the control of medical staff, and its constant level indicates that at an organizational level the emergency circuits were maintained completely functional. A small delay was indicated by Scholz et al. [12] in the time from arrival at the catheterization laboratory to vessel puncture (14.1 min before the pandemic and 12.9 min during) because of additional personal protection measures to avoid staff or patient contamination.

The total ischemic time was reported by three studies and increased from 229.48 ± 207.20 min to 417.06 ± 493.004 min during the first months of the pandemic, similar to symptoms-to-FMC time. The highest increase was indicated by Coughlan et al. [7], from a mean of 485 min to 1550 min. Even if not included in the meta-analysis, the studies of Reinstadler et al. [28] and Toner et al. [20] indicate a 1.7-fold and 3-fold increase in total ischemic times in Austria and Australia, respectively. The patient delay could be considered the most important factor driving the increase in total ischemic time as door-to-balloon time was maintained constant.

Six studies divided patients according to presentation delay [3,4,5,7,13,15]. Abdelaziz et al. [3] and Versaci et al. [15] reported that 26.10% and 16.67% of patients, respectively, presented more than 12 h after symptoms onset during the pandemic compared to none and 5.26% before. The threshold was considered 24 h by four studies used for the pooled analysis. Reported percentages for >24 h presentation delay varied between 4.3–27.8% before the pandemic to 21.7–44.22% during the outbreak. The pooled effect revealed that 34.40% of patients presented >24 h from symptoms onset during the outbreak compared to 15.33% before (Chi2 = 11.37, *p* < 0.001). Thus, during the pandemic, patients may pass from a time frame in which primary revascularisation provides substantial benefit to a time frame of minimal benefit and long-term morbidity and mortality, as the benefit of reperfusion in STEMI is time-dependent.

As a consequence of delayed presentation and prolonged total ischemic time, left ventricular impairment was more severe at admission, as shown by troponin-I levels and LVEF. Troponin-I levels registered higher values during the first months of the pandemic in three studies analysed [2,3,14]. The percentage of patients with low LVEF at admission (<40%), a strong predictor for 1-year mortality [29], was reported by Hammad et al., Popovic et al., and Secco et al. [2,10,13], and the pooled effect revealed a 2.25 higher risk of low LVEF at admission during the pandemic.

### 4.3. STEMI Outcome

In-hospital mortality was reported by eight studies and overall did not differ significantly between the two periods because of the increased contribution of the two large studies of Wu et al. [16] and Scholz et al. [12]. A single study performed in Italy by De Rosa et al. [8] on patients from 54 hospitals indicated an increase in the fatality rate from 4.1% to 11.9% for SARS-CoV-2 negative patients with STEMI. They also indicated an increase in major complications rate from 10.4% to 18.8%. The discrepancy between increased symptoms-to-FMC time, total ischemic time, number of patients presenting beyond the 24 h barrier since the onset of symptoms, and the overall stable mortality indicates that, despite the pandemic, health systems worldwide maintained a very high level of care and fully operational emergency circuits. Delays occur mostly at a patient level, and public awareness measures are necessary to reduce this patient-related delay.

Most patients survived the initial event, but as De Luca et al. [30] have stated, for each 30 min treatment delay the 1-year mortality increases by 7.5%. Early prognosis of STEMI patients presenting during the COVID-19 pandemic is favourable [31], but the real impact will be quantified by studies analysing mid- and long-term data, as the deleterious effect of late or no presentation could manifest in the following years by an increase in new heart failure cases and long-term cardiovascular mortality. In a study performed in 2021, Nioi et al. also indicated an increase in cases of medical liability due to delay in treatment/hospitalization for other diseases during the COVID-19 pandemic [32]. The societal aspect of the pandemic should also not be neglected by public health measures, as vulnerable populations are at higher risk of delayed treatment and suffer more from indirect consequences of the pandemic [33,34].

Mass-media involvement is necessary as the information spread during the initial stages of the pandemic concerning the lack of personal protection equipment in hospitals and medical systems focused on COVID-19 patients was not in favour of patients suffering from acute conditions [8,22]. Our study attempted an overview of the immediate impact of the COVID-19 pandemic on STEMI presentation and outcomes, but additional follow-up data are necessary for a complete picture.

### 4.4. Limitations

Our meta-analysis suffers from severe limitations. Firstly, it is based on a limited number of cross-sectional observational studies with a marked difference between sample sizes, evaluated periods, confounding factors, and contradictory results. The impact of bias on the estimated results is unknown. Secondly, we included uncontrolled studies performed all over the world during the early phases of the pandemic period in countries with different COVID-19 burdens and different social and healthcare measures (i.e., different hospital admission regulations, patient transport regulations, emergency stratification rules during lockdown periods, and organization of emergency medical services).

## 5. Conclusions

During the first months of the COVID-19 pandemic, a significant decrease in STEMI admissions was registered around the world. Patients presenting with STEMI were younger, with an increased comorbidity burden, were addressed later in the course of the disease, and had more severe left ventricular impairment. In-hospital emergency circuits and emergency care functioned properly, with no increase in door-to-balloon time nor in early mortality. Most delays registered were at a patient level. Healthcare systems should increase public awareness and encourage the seeking of medical services every time severe symptoms occur no matter the social or epidemiological context, as late presentation could increase long-term morbidity and mortality.

## Figures and Tables

**Table 1:** Summary of included studies.

No	Author	Type	No of Centres	Equivalent Time Periods	Groups	Number of STEMI Patients	SARS-CoV-2 Positive	Age, Years	Male, %
1	Abdelaziz et al. [3]	Retrospective cross-sectional observational (STEMI)	1 (UK)	Yes, 1–31 March 2019 1–31 March 2020	Pre-COVID COVID	69 46	No/Not indicated	66.6 ± 11.9 63.2 ± 11.1	76.8 69.6
2	Braiteh et al. [4]	Retrospective cross-sectional observational (ACS, STEMI extracted)	4 (NY, USA)	Yes, 1 March–30 April 2019 1 March–30 April 2020	Pre-COVID COVID	28 23	No/Not indicated	67.4 ± 16 58.6 ± 13	64.3 60.9
3	Tam et al. [5]	Retrospective cross-sectional observational (ACS, STEMI extracted)	1 (Hong-Kong)	No, 1 November 2019–24 January 2020 25 January 2020–31 March 2020	Pre-COVID COVID	36 27	-	Not specified for STEMI	Not specified for STEMI
4	Clayes et al. [6]	Retrospective cross-sectional observational (STEMI)	Multiple (Belgium, national registry)	No, 13 March–3 April 2017, 2018, 2019 13 March–3 April 2020	Pre-COVID COVID	761 188	7 (3.72%)—excluded from mortality	63 ± 15 63 ± 12	74 80
5	Coughlan et al. [7]	Retrospective cross-sectional observational (STEMI)	1 (Ireland)	Yes, 27 March–17 April 2019 27 March–17 April 2020	Pre-COVID COVID	14 9	No/Not indicated	59 ± 10 58 ± 17	100 55
6	De Rosa et al. [8]	Retrospective cross-sectional observational (ACS, STEMI extracted)	54 (Italy, national survey)	Yes, 12–19 March 2019 12–19 March 2020	Pre-COVID COVID	268 197	21 (10.7%)—excluded from mortality	65.4 ± 9.7 66.5 ± 10.2	75 79.69
7	Hammad et al. [2]	Retrospective cross-sectional observational (STEMI)	18 (OH, USA)	No, 1 January–22 March 2020 23 March 2020–15 April 2020	Pre-COVID COVID	108 35	No/Not indicated	61.8 ± 12.6 66 ± 10	72 49
8	Hauguel-Moreau et al. [9]	Retrospective cross-sectional observational (ACS, STEMI extracted)	1 (France)	No, 17 February–26 April 2018, 2019 17 February–26 April 2020	Pre-COVID COVID	63 16	1 (6.25%)	Not mentioned	Not mentioned
9	Popovic et al. [10]	Prospective cross-sectional observational (STEMI)	1 (France)	No, All patients 2008–2017 26 February–10.05.2020	Pre-COVID COVID	1552 72	No/Not indicated	59.6 ± 12.9 62.5 ± 12.6	76.10 73.60
10	Romaguera et al. [11]	Retrospective cross-sectional observational (STEMI)	10 (Spain)	Yes, 1 March–19 April 2019 1 March–19 April 2020	Pre-COVID COVID	524 395	No/Not indicated	63.4 ± 0.6 61.9 ± 0.7	79.20 80.25
11	Scholz et al. [12]	Retrospective cross-sectional observational (STEMI)	41 (Germany)	No, 1–31 March 2017–2019 1–31 March 2020	Pre-COVID COVID	1329 387	No/Not indicated	63.6 ± 0.4 64.5 ± 0.7	73 72
12	Secco et al. [13]	Retrospective cross-sectional observational (ACS, STEMI extracted)	3 (Italy)	Yes, 1–31 March 2019 1–31 March 2020	Pre-COVID COVID	59 34	Yes (number not mentioned for STEMI)	Not mentioned for STEMI	Not mentioned for STEMI
13	Tan et al. [14]	Retrospective cross-sectional observational (ACS, STEMI extracted)	1 (CA, USA)	No, 23 December 2019–18 March 2020 19 March–12 April 2020	Pre-COVID COVID	33 8	No/Not indicated	Not mentioned for STEMI	Not mentioned for STEMI
14	Versaci et al. [15]	Retrospective cross-sectional observational (STEMI)	1 (Italy)	Yes, 1–19 March 2019 1–19 March 2020	Pre-COVID COVID	38 24	No/Not indicated	Not mentioned	Not mentioned
15	Wu et al. [16]	Prospective cross-sectional observational (STEMI)	99 (UK)	No, 1 January 2019–22 March 2020 23 March–19 April 2020	Pre-COVID COVID	15,646 729	No/Not indicated	65.76 ± 13.44 64.64 ± 13.11	72 72

ACS—acute coronary syndromes; STEMI—ST-segment elevation myocardial infarction.

**Table 2:** Quality assessment using Newcastle–Ottawa scale.

No	Author	Selection				Comparability		Exposure			Score
		1	2	3	4	5.1	5.2	6	7	N.A.	
1	Abdelaziz et al. [3]	x	x	x	x	x		x	x		7
2	Braiteh et al. [4]	x	x	x	x	x		x	x		7
3	Tam et al. [5]	x		x	x	x		x	x		6
4	Clayes et al. [6]	x		x	x	x		x	x		6
5	Coughlan et al. [7]	x		x	x	x		x	x		6
6	De Rosa et al. [8]	x	x	x	x	x		x	x		7
7	Hammad et al. [2]	x	x	x	x	x		x	x		7
8	Hauguel-Moreau et al. [9]	x		x	x	x		x	x		6
9	Popovic et al. [10]	x	x	x	x	x		x	x		7
10	Romaguera et al. [11]	x	x	x	x	x		x	x		7
11	Scholz et al. [12]	x	x	x	x	x		x	x		7
12	Secco et al. [13]	x		x	x	x		x	x		6
13	Tan et al. [14]	x		x	x	x		x	x		6
14	Versaci et al. [15]	x		x	x	x		x	x		6
15	Wu et al. [16]	x	x	x	x	x		x	x		7

N.A.—not applicable.

**Table 3:** Patient baseline characteristics.

	Pre-COVID-19	COVID-19	*p*
Age (mean ± SD) (10 studies)	64.96 ± 12.90	63.98 ± 9.9	0.0008
Male patients (n, %) (10 studies)	14,732 (72.58)	1545 (74.24)	0.103
Arterial hypertension (n, %) (7 studies)	7942 (40.77)	660 (45.02)	0.00143
Diabetes mellitus (n, %) (7 studies)	3662 (18.80)	307 (20.94)	<0.001
Smoking (n, %) (6 studies)	6239 (33.33)	452 (35.36)	<0.001
Dyslipidaemia (n, %) (5 studies)	4576 (24.59)	3232 (25.98)	<0.001
Family history (n, %) (3 studies)	242 (17.14%)	88 (19.91%)	0.18
Known coronary artery disease (n, %) (3 studies)	229 (11.91)	102 (12.32)	<0.001

SD—standard deviation.
